# Integration of a Dielectrophoretic Tapered Aluminum Microelectrode Array with a Flow Focusing Technique

**DOI:** 10.3390/s21154957

**Published:** 2021-07-21

**Authors:** Naqib Fuad Abd Rashid, Revathy Deivasigamani, M. F. Mohd Razip Wee, Azrul Azlan Hamzah, Muhamad Ramdzan Buyong

**Affiliations:** Institute of Microengineering and Nanoelectronics (IMEN), Universiti Kebangsaan Malaysia (UKM), Bangi 43600, Selangor, Malaysia; p103412@siswa.ukm.edu.my (N.F.A.R.); p101094@siswa.ukm.edu.my (R.D.); m.farhanulhakim@ukm.edu.my (M.F.M.R.W.); azlanhamzah@ukm.edu.my (A.A.H.)

**Keywords:** tapered aluminum microelectrode array, dielectrophoresis force, hydrodynamic flow focusing, microfluidics

## Abstract

We present the integration of a flow focusing microfluidic device in a dielectrophoretic application that based on a tapered aluminum microelectrode array (TAMA). The characterization and optimization method of microfluidic geometry performs the hydrodynamic flow focusing on the channel. The sample fluids are hydrodynamically focused into the region of interest (ROI) where the dielectrophoresis force (F_DEP_) is dominant. The device geometry is designed using 3D CAD software and fabricated using the micro-milling process combined with soft lithography using PDMS. The flow simulation is achieved using COMSOL Multiphysics 5.5 to study the effect of the flow rate ratio between the sample fluids (Q_1_) and the sheath fluids (Q_2_) toward the width of flow focusing. Five different flow rate ratios (Q_1_/Q_2_) are recorded in this experiment, which are 0.2, 0.4, 0.6, 0.8 and 1.0. The width of flow focusing is increased linearly with the flow rate ratio (Q_1_/Q_2_) for both the simulation and the experiment. At the highest flow rate ratio (Q_1_/Q_2_ = 1), the width of flow focusing is obtained at 638.66 µm and at the lowest flow rate ratio (Q_1_/Q_2_ = 0.2), the width of flow focusing is obtained at 226.03 µm. As a result, the flow focusing effect is able to reduce the dispersion of the particles in the microelectrode from 2000 µm to 226.03 µm toward the ROI. The significance of flow focusing on the separation of particles is studied using 10 and 1 µm polystyrene beads by applying a non-uniform electrical field to the TAMA at 10 V_PP_, 150 kHz. Ultimately, we are able to manipulate the trajectories of two different types of particles in the channel. For further validation, the focusing of 3.2 µm polystyrene beads within the dominant F_DEP_ results in an enhanced manipulation efficiency from 20% to 80% in the ROI.

## 1. Introduction

### 1.1. Microfluidic Technology for Biological Analysis

The manipulation and separation of targeted particles from their sample play an essential role in biomedical analysis. Recent developments in microfluidic technology and the manipulation and separation of particles can be achieved on a chip. The advantages of this method are portability, only a small amount of sample is required, low cost and low time consumption compared with the conventional method [1,2]. The difference in size, density [3,4], magnetic properties [5] and dielectric properties [6,7] can manipulate the targeted particles from their sample.

**Figure 1:**
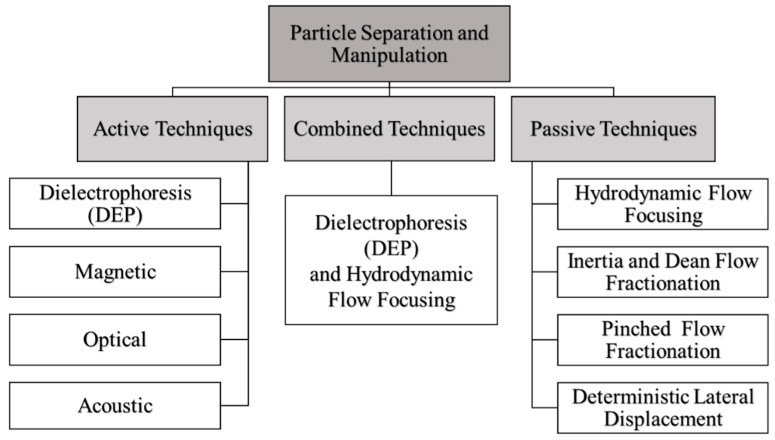
A broad classification of various microfluidic separation techniques.

Microfluidic separation can be classified into three categories, which are active, passive and combined techniques, as can be seen in Figure 1. Active techniques use an external field to manipulate the movement of the particle while the passive technique involves the interaction between the microchannel structure and the flow field to manipulate the particles. To support the present work, a hydrodynamic flow focusing technique, Tanyeri et al. developed a focusing channel inlet that is identical to the inlet used in this study to achieve high manipulative outcomes [8,9,10]. According to Gossett et al., the microfluidic devices were fabricated using standard photolithographic methods and they used the SU-8 mold, which is easily damaged [11]. However, we used a 3D printing mold or computer numerical control (CNC) milling process, which have the advantage of being able to be used multiple times without causing damage. Furthermore, in this current work, we integrated with a tapered aluminum microelectrode array (TAMA) to reduce the dispersion width and improve the particle manipulation efficiency.

Alazzam et al. proposed a microfluidic device capable of separating green fluorescent protein-labeled MDA-MB-231 and other types of cancer cells from healthy ones using planar interdigitated dielectrophoresis (DEP) microelectrodes [12]. Chen et al. designed and fabricated an ICEO microfluidic chip that combined DEP to separate particles with different dielectric properties of yeast cells from silica particles continuously [13]. Li et al. created a miniature isomotive DEP-based continuous cell-sorting device integrated with a microfluidic chip to distinguish between living and dead yeast cells [14]. Yang et al. demonstrated the development of a DEP microfluidic device with interdigitated microelectrodes to concentrate and selectively capture listeria cells bonding with antibodies from other types of cells [15]. Choi et al. demonstrated a microfluidic device for DEP based on a trapezoidal electrode array (TEA) for separating different sizes of polystyrene beads [16]. In 2009, Wang et al. developed a microfluidic DEP separation device with interdigitated microelectrodes in the sidewalls of microchannels for the separation of beads and cells [17]. The recent trends in incorporating the microfluidic principle with the DEP technique as well as their applications are discussed. However, none of them focus on the TAMA profile microelectrode. They cover different aspects of various designs. Moreover, our study concentrates on the TAMA profile microelectrode.

Active sorting techniques such as DEP are among the most familiar separation techniques that have been studied in recent research [18,19,20]. DEP is a method that utilizes the dielectric properties of particles [21]. In this technique, different dielectric particles can be manipulated by implementing a sinusoidal time-varying and spatially non-uniform electrical field. Particles that are more polarizable than the suspended medium will move toward the region of the strong electrical field and such a movement is called positive dielectrophoresis (P_DEP_). In contrast, the particles that are less polarizable than the suspended medium will move toward a low electrical field. This motion is referred as negative dielectrophoresis (N_DEP_) [22,23,24,25,26]. This scenario plays an important role in the biomedical analysis as it depends on the success of the separation and isolation of particles from their main sample.

In this study, a TAMA profile microelectrode is used which in turn generates a non-uniform electric field for a DEP force (F_DEP_). A TAMA consists of an aluminum microelectrode array on a silicon substrate and is fabricated based on the CMOS processing technique [27,28]. Based on the investigation from our previous studies, the F_DEP_ produced by this microelectrode produced a higher gradient non-uniform electrical field from the top and bottom edges of the microelectrode [29,30]. It managed to separate and isolate more than one type of particle depending on the F_DEP_ acting on the particles by adjusting the frequency of the electrical field.

Through a TAMA type of microelectrode, the manipulation of targeted particles has only been studied in the gap between two edges of microelectrodes called the region of interest (ROI) [31]. At this region, the magnitude of the F_DEP_ is dominant and strong enough to influence the movement of the particle while in the other region, the fluid-particle interaction will be dominated by the fluid flow consequently affecting the DEP effect on the particles [32]. Thus, it faces a challenging problem to manipulate all of the particles as the particles will disperse all around the microelectrodes once it is fed to the channel.

As a solution to this problem, we proposed the integration of a hydrodynamic flow focusing technique to reduce the particle dispersion that involves a TAMA in an F_DEP_ application. There are different unique microelectrode designs with flow focusing combinations that have been reported [33,34,35]. According to Shkolnikov et al., the manipulation of particles has achieved an 80% separation efficiency by the integration of the F_DEP_ and the hydrodynamic flow focusing technique [36]. 

In this work, various techniques for a TAMA interaction with flow focusing are examined by introducing a hydrodynamic flow focusing of the sample to the TAMA profile microelectrode wall. The F_DEP_ strives to push the targeted particles away from the sample fluid to the sheath fluid and separates them from the main sample by introducing a flow focusing to the wall of the microelectrodes. We focus on the characterization and optimization of a hydrodynamic flow focusing technique on a TAMA DEP design application.

### 1.2. Active Technique: DEP

The electrical polarizable technique experiences a force due to the interaction of electrical properties between the particle and the medium. The active technique of particle separation using DEP is given by the equation below:

(1) $$F_{DEP}=2\pi \varepsilon_{o}\varepsilon_{medium}r^{3}\;Re\;CMF\nabla E^{2}$$

where *ε_o_* is the permittivity for the vacuum 8.854 × 10^−12^ F/m and *ε_medium_* is the relative permittivity of the suspended medium. The Clausius–Mossoti factor (CMF) is the frequency-dependent reaction and is formulated by:

(2) $$\mathrm{CMF}=\frac{\left(\varepsilon^{*}{}_{particle}-\varepsilon^{*}{}_{medium}\right)}{\left(\varepsilon^{*}{}_{particle}+2\varepsilon^{*}{}_{medium}\right)}$$

where

(3) $$\varepsilon^{*}{}_{particle\;}=\varepsilon_{particle}-\frac{j\sigma_{particle}}{\omega }$$

and

(4) $$\varepsilon^{*}{}_{medium}=\varepsilon_{medium}-\frac{j\sigma_{medium}}{\omega }$$

where the ε*_particle_ is the complex permittivity of the particle, *ε_particle_* is the absolute permittivity of the particle, *σ_particle_* is the conductivity of the particle and *σ_medium_* represents the conductivity of the medium.

### 1.3. Passive Technique: Hydrodynamic Flow Focusing

The flow of fluid follows two general regimes of laminar and turbulent, which are considered by the comparative significance of the inertial to viscous forces explained by the Reynolds number (*R_e_*). At a low *R_e_*, the flow is laminar, which flows parallel to each other and mixes only through convection and diffusion. A flow with a high *R_e_* is a chaotic flow in which the fluid undergoes irregular fluctuations or mixing in contrast to a laminar flow. The change among laminar and turbulence flows, in general, happens above Reynolds 2000 in interior flows. The *R_e_* is explained as:

(5) $$R_{e}=\frac{\rho VL}{\mu }$$

where ρ is the fluid density, *V* is the average velocity, *L* is the length scale and *μ* is the fluid velocity.

**Figure 2:**
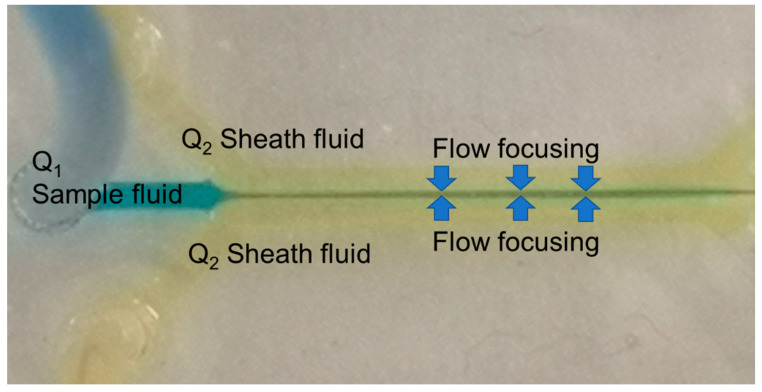
Hydrodynamic flow focusing microfluidic channel.

Theoretically, the microscale dimensions of the flow in microfluidics inhibits the laminar regime because of the small Reynolds number. This advantage can lead to a hydrodynamic flow focusing technique. Hydrodynamic flow focusing is a technique in which a sheath fluid is introduced from the side to the side of the main flow to squeeze the flow sample [34]. The inability of the sample fluid to mix with the sheath fluid in the laminar flow region is the reason for producing flow-focusing with a different flow rate ratio. where the fluid flow is described by parallel lines flowing linearly with no mixing as a very ordered flow. As shown in Figure 2, the sample fluid Q_1_ is focused and sheathed downstream by sheath flow Q_2_ [35]. The size of flow focusing is tuned along the main channel by characterizing and optimizing the flow rate ratio of the particles to the ROI where the F_DEP_ is dominant. 

## 2. Materials and Methods

**Figure 3:**
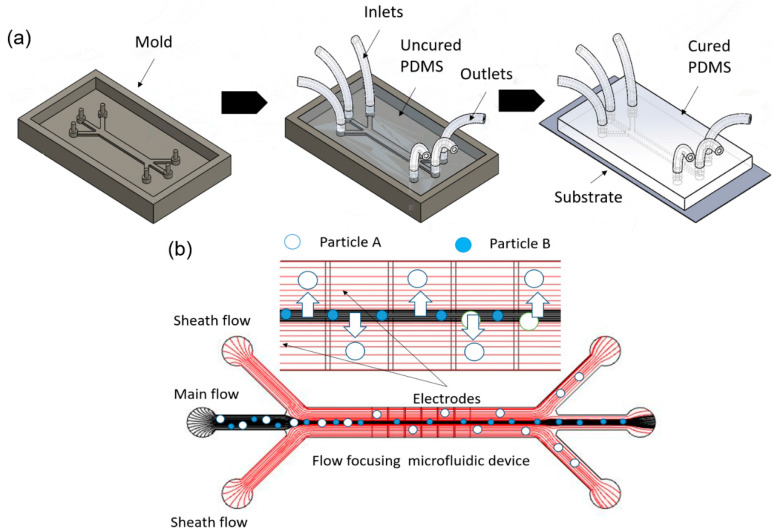
(**a**) Microfluidic device fabrication process: molding, casting and bonding; (**b**) flow focusing design.

The material used in this study includes fluorescent polymer microspheres, 1, 3.2 and 10 µm, from Thermo Fisher Scientific (Fluoro-max Dyed, Thermo Fisher, Scientific Inc., Waltham, MA, USA). We used polydimethylsiloxane (PDMS) as a microfluidic layer because of its transparency. A Sylgard 184 silicone elastomer base and curing agent from the Dow Corning Corporation (Midland, MI, USA) were used to produce a PDMS microfluidic channel. The PDMS base and curing agent were combined in a 10:1 mass ratio before pouring into the mold. Afterward, the microfluidic layer and the TAMA profile microelectrode substrate were bonded using oxygen plasma. The process of molding, casting and bonding are illustrated in Figure 3a. 

### 2.1. Device Design

We used 3D CAD software (SolidWorks 2020, accessed on 1 October 2019) to design our channel. A schematic diagram of the proposed design is shown in Figure 3b based on a TAMA profile microelectrode designed with 24 pairs. Each microelectrode dimension was 1000 × 1000 µm with a microelectrode gap of 80 µm as the ROI [36]. The flow focusing microfluidic device consisted of three inlets and three outlets. The two side inlets were used as a sheath flow while the middle inlet was used for the main flow. The inlets and outlets then created three flow streams in the channel. In characterizing and optimizing the flow rate of the main flow, we could tune the width of the main flow to the center of the TAMA profile microelectrodes.

The microfluidic device was fabricated using a soft lithography technique. The formation of an SU-8 mold was replaced by 3D printing or a CNC milling process. The benefits of using this process were that it facilitated a precise design control and a robust mold compared with the SU-8 mold and it could be reused. The steps involved a casting process of a polymer material to form a mold. An assembly process then took place as the parts were cast separately. This assembly process involved an alignment and stacking procedure. The parts were joined together with a bonding technique. The depth of the channel was 500 µm. Once the mold was designed, it was then transferred to the software used for the CNC milling machine (Roland MDX-40a). The PDMS layer contained a microchannel that held the liquid sample and allowed the fluid to flow from the inlet to the outlet. The microelectrode layer at the bottom of the PDMS layer served as a substrate for particle manipulation in the channel.

### 2.2. Flow Simulation

The characterization and optimization of a suitable flow rate ratio by a numerical simulation study were achieved by using COMSOL Multiphysics 5.5 (www.comsol.com, accessed on 14 November 2019). A theoretical volumetric flow rate ratio was studied to obtain the desired width of flow focusing. The simulation was modeled under the conditions of a laminar flow and the transport of diluted species using a 3D model. The model was computed under a stationary time. The device geometry and the flow rate parameters were set to be identical to the real devices and experiment.

### 2.3. Experimental Setup

In the flow focusing preliminary experimental setup, two ink solutions were used. The yellow-colored deionized (DI) water was used as a sheath fluid while blue-colored DI water was used for the sample fluid to identify and characterize the ability of hydrodynamic flow focusing. The chip that was connected to the microfluidic tubing (Tygon tube) was inserted into the inlets and outlets (middle outlet) for the pumping process. The fluid was then pumped into the system through the inlets using a Terumo syringe (Tuberculin, 1 mL) and a syringe pump system (Longerpump, TS-2A/L0107-2A).

The flow rates of the sample fluid (Q_1_) were manipulated at 500, 400, 300, 200 and 100 μL/m while the flow rate of the sheath fluid (Q_2_) was kept constant at 500 μL/m. The device was manually loaded with DI water to pre-fill all of the fluidic channels to eliminate any trapped air bubbles in the microchannel. Air bubbles, for example, which can be present at the inlet ports, could disrupt hydrodynamic focusing and therefore impact on the flow of the sample stream while air bubbles at the outlet ports could adversely impact the operation reliability and the effectiveness of the device in producing flow focusing. In this experiment, a glass slide was used as a substrate.

**Figure 4:**
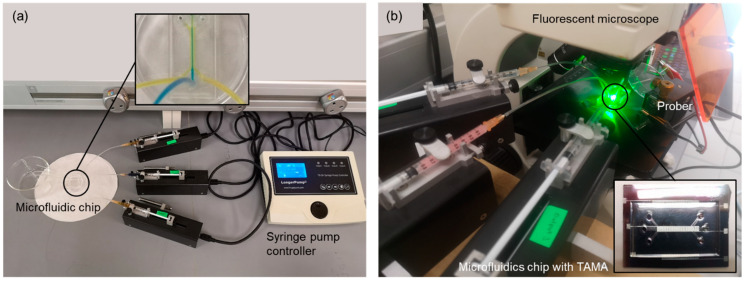
Experimental setup for (**a**) leaking test; (**b**) DEP experimental setup of the integrated TAMA and microfluidics.

A secondary experimental validation of the focusing performance was conducted via the monitoring of the top view of the flow pattern inside the microchannel using a standard camera, as shown in Figure 4a, to observe the significance of the flow focusing technique on improving DEP separation in the TAMA. A detailed experiment was set up by bonding the device with the TAMA profile microelectrode. The manipulation of 3.2 μm polystyrene beads was used for visualizing the particle movement in the flow focusing effect while the buffer syringe was filled with pure filtered DI water. 

For the ultimate experimental validation of the DEP particle separation, 10 and 1 μm polystyrene beads were used. The system consisted of an additional alternating current (AC) power supply generator (Teledyne LeCroy-WaveStation 2022–20 V peak to peak (V_PP_), 15 MHz) with a connected prober to control the current with the specific frequency applied to the microelectrodes. The experiments were performed at 10 V_PP_ and the frequency of the supplied voltage was set to 150 kHz. A frequency of 150 kHz was selected.

As a result, the 10 μm polystyrene beads would experience N_DEP_ while 1 μm particles would experience P_DEP_, based on previous studies [31,37]. The image was captured by a microscope with a built-in camera (Olympus BX53M). The detail of the experiment is shown in Figure 4b.

## 3. Results

### 3.1. Leaking Test

Within the evaluation of the functionality of the fabricated microfluidic device in producing flow focusing a leaking test was conducted. In this investigation, five different flow rate ratios between the main flow and the sheath flow (Q_1_/Q_2_) were set up to observe the presence of any leakage within the microfluidic channel. The conditioning fluid flow was also observed to determine if it flowed in laminar or turbulence. The flow rate ratios in this experiment were set up at 0.2, 0.4, 0.6, 0.8 and 1.0.

**Figure 5:**
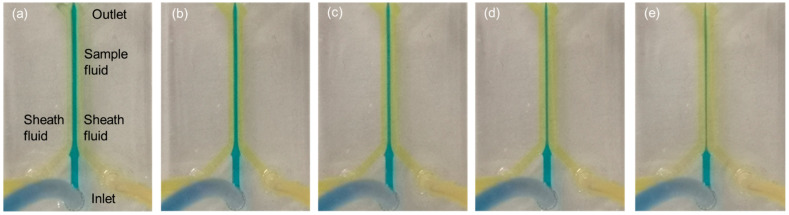
Flow focusing effects with five different flow rate ratios: (**a**) 1.0; (**b**) 0.8; (**c**) 0.6; (**d**) 0.4; (**e**) 0.2.

Based on Figure 5a–e, it could be concluded that the fluid flow in all conditions was ideal, which indicated that there was no blockage in the microchannel or any leakage between the PDMS layer and the glass slide. This microfluidic chip also demonstrated its ability to produce a laminar flow profile as flow focusing effects were obtained for all of the parameters, which implied there was no mixing occurring between the main flow and the sheath flow.

### 3.2. Effect of the Flow Rate Ratio to the Hydrodynamic Flow Focusing Width

The capability of producing flow focusing on the microelectrodes was evaluated by the microfluidic chip bonded to the TAMA. The width of flow focusing was analyzed by using five different flow rate ratios between the main flow and the sheath flow (Q_1_/Q_2_). The width of flow focusing was analyzed by measuring the focused streamline at the center along the channel. The width of flow focusing was then measured by using imaging software (Olympus CellSens-Standard).

**Figure 6:**
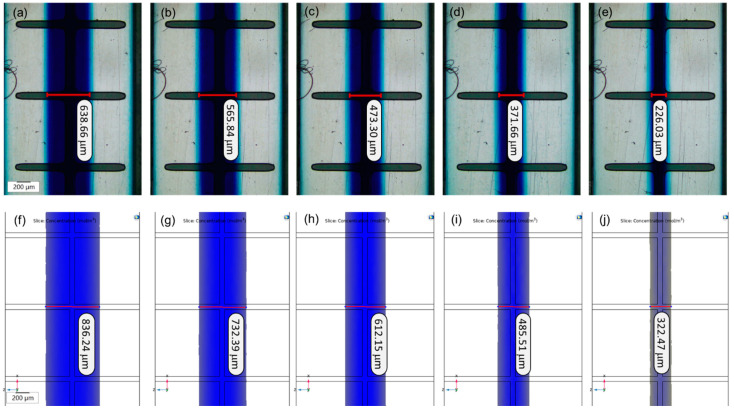
The flow focusing width obtained in the TAMA toward 1.0, 0.8, 0.6, 0.4, 0.2; (**a**–**e**) experimental (**f**–**j**) simulation.

Subsequently, the experimental results were compared with the COMSOL simulation. The width of flow focusing was measured using the concentration effect for the simulation results. The method for the measurement of the width of flow focusing for both the experiment and the simulation can be seen in Figure 6a−e and Figure 6f−j, respectively. The results of the width of flow focusing with different flow rate ratios are tabulated in Table 1.

**Figure 7:**
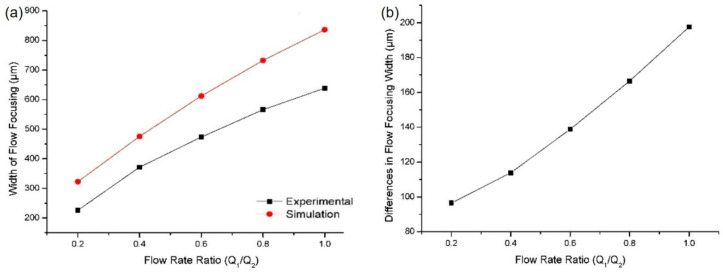
(**a**) Flow focusing width at different flow rate ratios; (**b**) flow focusing width differences between the simulation and the experiment at different flow rate ratios.

From Table 1, it was revealed that the width of flow focusing increased with the flow rate ratio for both the experiment and the simulation. The flow focusing width between the experimental and simulation trends can be seen from the graph plotted in Figure 7a. The graph for the experiment and the simulation shows the same trend and these results were expected based on previous studies because as the main flow increases, the main fluid starts to dominate the channel [32,38,39,40,41,42]. As the flow rate ratio decreases between the main and the sheath flow, the main fluid is less dominant in the channel resulting in the reduction of the flow focusing width.

However, the width differences between the experiment and the simulation increased with the flow rate ratio, as can be seen in the graph in Figure 7b. At Q_1_/Q_2_ = 0.2, the width difference was only about 96.44 µm before it increased to 197.58 µm at Q_1_/Q_2_ = 1.0. This was due to the inaccurate flow rate value between the experiment and the simulation as the flow rate fluctuations came from the mechanical syringe pump to feed the fluid to the channel. Flow rate fluctuations occurred in the syringe pump-driven system because of the mechanical oscillation of the pump motor and it was not precisely calibrated. There might also have been an inaccuracy in the reading of the flow rate value in the system.

Consequently, the flow rate value between the experiment and the simulation may not be accurate, resulting in differences in the flow focusing width. In this research, these differences in value between the experiment and the simulation did not have a significant impact on the particle separation. From these results, at a 0.2 flow rate ratio between the main flow and the sheath flow, we reduced the width of the main sample in the channel by a 10:1 ratio from 2000 µm to 226.03 µm.

After successfully producing flow focusing on the channel, the dispersion of the particles was studied using polystyrene beads as an interest in observing the ability of flow focusing to reduce the dispersion of the particles in the TAMA. The experiment was repeated three times by feeding the polystyrene beads to the main sample and the flow rate ratio between the main flow and the sheath flow was set up at 0.2. The movement of the particles was recorded under a fluorescence light source and 3.2 µm particles were used in this experiment to provide a better visualization.

**Figure 8:**
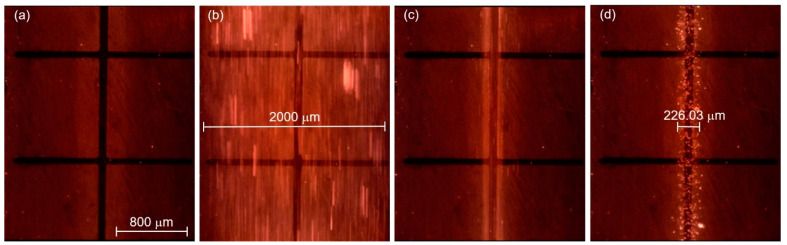
Particle movement: (**a**) particles before entering the channel; (**b**) particles entering the channel; (**c**) the particles were focused once the sheath flow was introduced; (**d**) particles contained within the ROI.

The movement of the particles is depicted in Figure 8 with four different phases. During the first phase, Figure 8a, the TAMA profile microelectrode was shown without the flow of the particle. The main flow containing particles was then fed to the microfluidic chip without any sheath flow interference and is presented in Figure 8b. In this phase, the particles were dispersed in all of the surfaces of the microelectrodes. As shown in Figure 8c, once the sheath flows were introduced, the dispersion particles were squeezed by both sheath fluids resulting in the particles focusing on the ROI. The flow was then stopped to manipulate or separate the particles in the ROI, as shown in Figure 8d and its recording is given in Video S1. It could be concluded that the particles remained in the main sample after the sheath flow was initiated. With a repeated analysis, the particle dispersion was significantly reduced from 2000 µm to 226.03 µm at a 0.2 flow rate ratio between the main flow and the sheath flow.

### 3.3. DEP Separation

The significance controlling the dispersion width of the particles on the TAMA at the DEP of the ROI area was studied by separation of 10 µm and 1 µm polystyrene beads in the DI water medium. The TAMA was supplied at a constant applied 10 V_PP_ with a frequency of 150 kHz for three minutes. The chosen value of the frequency applied was based on a CMF calculation and exposed the used particles for two different F_DEPs_. The 10 µm was exposed to a vertical repelled under a negative F_DEP_, N_DEP_ and the 1 µm was exposed to a laterally attracted under positive F_DEP_ and P_DEP_ subjected to a high electric field region.

The trajectories for both particles were observed under a fluorescence light source with three different filters to view each specific size of the particles. A UV filter was used to observe both particle trajectories while the red, blue and green filters were used to observe the 10 and 1 µm particles. The red and blue filters were used specifically for the 10 µm green particle observation while the blue and green filters were used for the 1 µm red particles.

The magnified views of the microelectrodes at 10× are presented in the figures in Table 2. The capturing action was performed as per the following time frame. Initially, all of the particles were focused by hydrodynamic flow focusing within the ROI as shown in Table 2 figures a, c and e. The particles were then manipulated and separated by applying F_DEP_ and the trajectories were tracked for three minutes as shown in Table 2 figures b, d and f. From the preliminary observation, the ability of the DEP integration with the hydrodynamic flow focusing of a microfluidic device in separation was achieved for all of the particles in the ROI.

In Table 2 figure b, it is shown that the 10 µm particles were vertically repelled with N_DEP_ in two different directions depending on the position of the particle. From the experimental observation, the particles that were situated at the ROI tended to move to the center of the gap between the two microelectrodes while the particles that were situated at the surface of the microelectrode slightly away from the ROI tended to move away from the edges of the microelectrodes. Both movements show the N_DEP_ movement where it was repelled from the high electric field region.

Based on the simulation results and experimental works done by Buyong et al. [31], the highest electrical field region was situated at the edge of the microelectrode. Due to the strong electrical field that presented in the edges of the microelectrodes, the 10 µm particles in the gap between the two-edge microelectrode, the ROI in Table 2 figure c tended to move to the center as the region had a relatively lower electrical field compared with the edges of the microelectrode. These edges acted as a barrier that prevented the particles in the center from moving to the side of the microelectrode resulting in two different movements of the 10 µm particles because of the position of the particle. A detailed movement of the 10 µm particles can be seen in Table 2 figures c and d.

The trajectory of the 1 µm particle movements were lateral attracted, with P_DEP_ to the higher electrical field region, which was situated at the edges of the microelectrode. The particle size was too small compared with the 10 µm particles to barely observe the movement under the UV filter. Thus, the movement of the 1 µm particles could only be seen clearly under the blue and green filters, as shown in Table 2, figure e and f. As shown in the illustration in the Table, 1 µm red particles were initially dispersed within the ROI. After 10 V_PP_ with a frequency of 150 kHz was applied, the particles were laterally attracted to the edges of the microelectrode where a high electrical field was situated showing the P_DEP_ movement and is presented in Table 2, figure f.

## 4. Discussion

The validation of the integration of a DEP TAMA with a flow focusing technique was specified into four analysis: (a) a leaking test and flow focusing test of yellow and blue dye, (b) a flow focusing test of 3.2 µm, (c) a DEP and flow focusing test of 1 and 10 µm for a manipulation and separation application and (d) a DEP and flow focusing test of 3.2 µm for a manipulation efficiency analysis.

**Figure 9:**
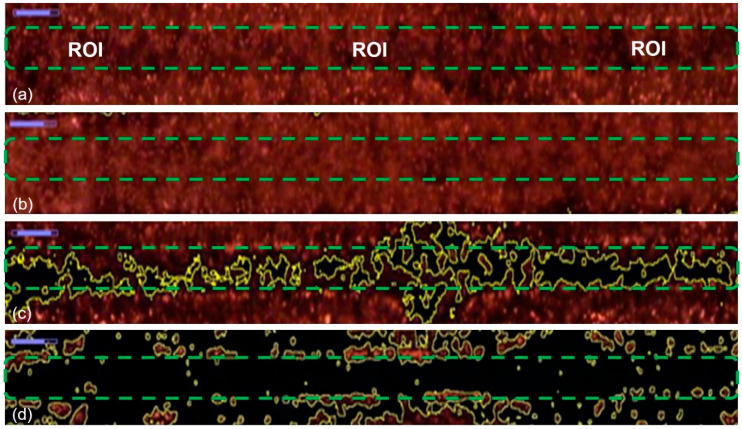
Particle surface area the red color indicates 3.2 µm particle, black color represents absence of particle: (**a**) before DEP force without flow focusing; (**b**) before DEP force with the sheath flow; (**c**) after DEP force without the sheath flow; (**d**) after DEP force with the sheath flow.

The ultimate validation was the manipulation efficiency and was analyzed in the area of the 3.2 µm particles covered in the ROI. The condition before and after DEP manipulation and integration without and with flow focusing were demonstrated. Using an ImageJ analysis, Figure 9 shows that the area of the 3.2 µm particle was measured within the ROI. The efficiency enhancement of manipulation via the combination of flow focusing and F_DEP_ is elaborated in the Supplementary Materials.

The principle of integration was flow focusing the particle to the ROI by concentrating the dispersion of the particle exposed to F_DEP_. The manipulation of the particles under lateral attraction P_DEP_ then forced the particle to migrate from the sample fluid to the sheath fluid. The continuous flow focusing enhanced the lateral attraction P_DEP_ of the particle due to the reduction of the loading of the F_DEP_ manipulation in the ROI. The measurement was taken five times for each condition. The values are tabulated in Table 3 and detailed calculations are given in the Supplementary Materials.

As depicted in Table 3, the efficiency of the manipulation of 3.2 µm particles without the flow focusing condition was achieved at 20% while in the flow focusing condition, the efficiency of the particle separation was achieved at 80%. A significant correlation of the four analyses of the integration of the DEP TAMA with the flow focusing technique was able to enhance the particle manipulation efficiency in the TAMA type microelectrode application.

## 5. Conclusions

This research work aimed to advance the DEP mechanism, which is specific in terms of particle manipulation and separation efficiency for a TAMA profile microelectrode application. The identified root cause was due to the dispersion of particles out from the DEP ROI range. The utmost solution was the reduction of the dispersion of the particles in the microfluidic channel using the flow focusing technique. The microfluidic chip was fabricated using a micro-milling technique assisted with a soft lithography PDMS, which is one of the low-cost techniques. The ability to produce flow focusing on the ROI was obtained with five different flow ratios between the main flow and the sheath flow (Q_1_/Q_2_). The outcome, the integration of a flow focusing microfluidic chip with a TAMA DEP microelectrode, reduced the particle dispersion from 2000 µm to 226.03 µm, resulting in enhancing the particle separation efficiency from 20% to 80%.

## Figures and Tables

**Table 1:** Flow focusing width for experimental and simulation results.

Flow Rate Ratio between the Main Flow and the Sheath Flow (Q1/Q2)	Experimental Flow Focusing Width (µm)	Simulation Flow Focusing Width (µm)	Differences (µm)
0.2	226.03	322.47	96.44
0.4	371.66	485.51	113.85
0.6	473.30	612.15	138.85
0.8	565.84	732.39	166.55
1.0	638.66	836.24	197.58

**Table 2:** The manipulation of particles in a fluorescence light source with three different light filters: (**a**,**b**) UV filter; (**c**,**d**) red-blue filters; (**e**,**f**) blue-green light.

Figures	Mechanism
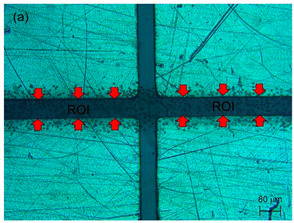	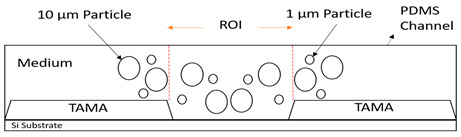
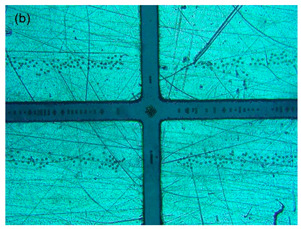	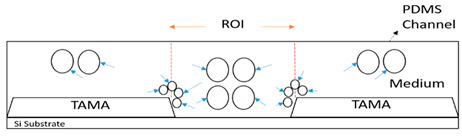
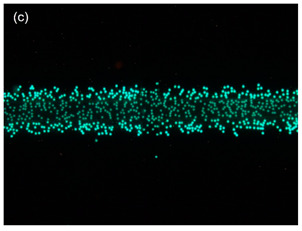	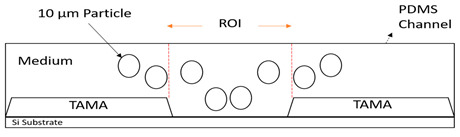
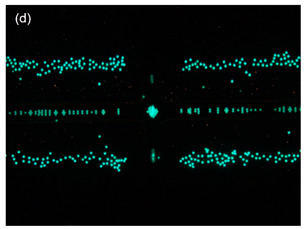	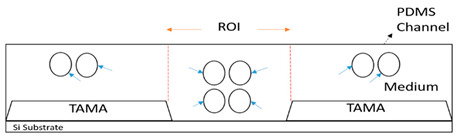
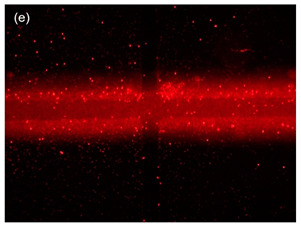	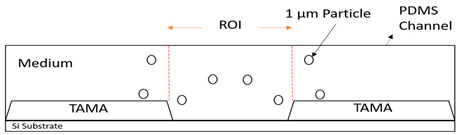
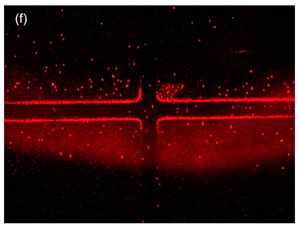	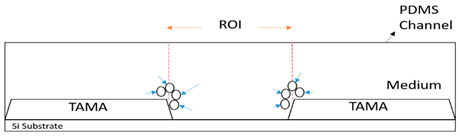

**Table 3:** The efficiency of manipulating a 3.2 µm polystyrene particle with and without flow focusing.

Condition	Before Manipulation (µm^2^)	After Manipulation (µm^2^)	Manipulation Efficiency (%)
Area covered by particles without flow focusing	519,422.8	414,208.1	20.3
Area covered by particles with flow focusing	517,465.7	102,116.3	80.3

## Data Availability

The data presented in this study are available within this article.

## Supplementary Materials

The following are available online at https://www.mdpi.com/article/10.3390/s21154957/s1, Video S1: The supplementary text document includes the efficiency enhancement of manipulation via the combination of flow focusing and F_DEP_ and the manipulation efficiency calculation in Section 4.
