# Supplementary material for: Integration of a Dielectrophoretic Tapered Aluminum Microelectrode Array with a Flow Focusing Technique
Source: Sensors (Basel). 2021 Jul 21;21(15):4957. doi: 10.3390/s21154957 (PMC8347692; doi:10.3390/s21154957)
Supplement: Supplementary file 1 [file sensors-21-04957-s001.zip › sensors-1268397-supplementary.pdf]

Supplementary Information

Integration of Dielectrophoretic Tapered Aluminum Microelectrode Array with Flow Focusing Technique

N.F. Abd Rashid [1], Revathy Deivasigamani [1], M.F. Mohd Razip Wee [1], Azrul Azlan Hamzah [1], and Muhamad Ramdzan Buyong [1, *]

[1]Institute of Microengineering and Nanoelectronics (IMEN), Universiti Kebangsaan Malaysia (UKM), 43600 Bangi, Selangor, Malaysia; p103412@siswa.ukm.edu.my (N.F.); p101094@siswa.ukm.edu.my (R.D); m.farhanulhakim@ukm.edu.my (M.F.); azlanhamzah@ukm.edu.my (A.A.); muhdramdzan@ukm.edu.my (M.R.)

* Correspondence: muhdramdzan@ukm.edu.my (M.R.); Tel.: +60-12-385-2713

Enhance efficiency of separation or manipulation via the combination of Flow-focusing and F_{DEP}:

- The following method is used to enhance separation or manipulation efficiency by combining flow-focusing and F_{DEP}. Observation from the experimental, without the flow-focusing the width of the particle dispersion approximately 2000 µm. Implementation of the flow-focusing reduced the width of the particle dispersion approximately to 226.03 µm.

- Without the flow focusing, the separation or manipulation efficiency is 20.3 %. Further enhancement of efficiency of approximately 80.3 % by introducing the flow-focusing resulted in reduced the width of the particle dispersion approximately to 226.03 µm.

Table S1. Manipulation efficiency and flow width with and without flow-focusing.

Condition	Sample flow width (µm)	Efficiency (%)
Without flow-focusing	2000	20.3
With flow-focusing	226.03	80.3

- The flow focusing may lead to a decrease in the volume of the particle dispersion. However, flow-focusing contribute to higher efficiency due to the flow focusing mechanism that concentrates the particle into the ROI. The width of the particle dispersion reduced from 2000 to 226.03 µm, where F_{DEP} is dominant. This is because the efficiency is calculated based on the particle present in the ROI.

- Flow focusing may reduce particle flow volume, but it is not a dominant parameter. The most important is the higher particle density in ROI. This is because to enhance F_{DEP} exposed to the

particle at ROI. The particle dispersion out of ROI (2000 μm) is not affected by F_{DEP} as compared to the particle dispersion with the flow-focusing (226.03 μm). The F_{DEP} is strong enough to separate or manipulate the particle as long as it is in ROI.

Explanation and calculation of Manipulation Efficiency (%) presented in table 3.

Define

- Figure 9 (In article) shows how the area of the particle was measured within the ROI using ImageJ analysis. Each condition was measured three times.

- The Figure S1 below shows the details, we define the area in ROI by the length of ImageJ analysis approximately 2300 μm and the width approximately 226.03 μm. Thus, the total area of ROI ImageJ analysis 519,869 μm^2. However, ImageJ analysis total area 519,422.8 μm^2 (Without flow focusing) and 517,465.7 μm^2 (with flow focusing).

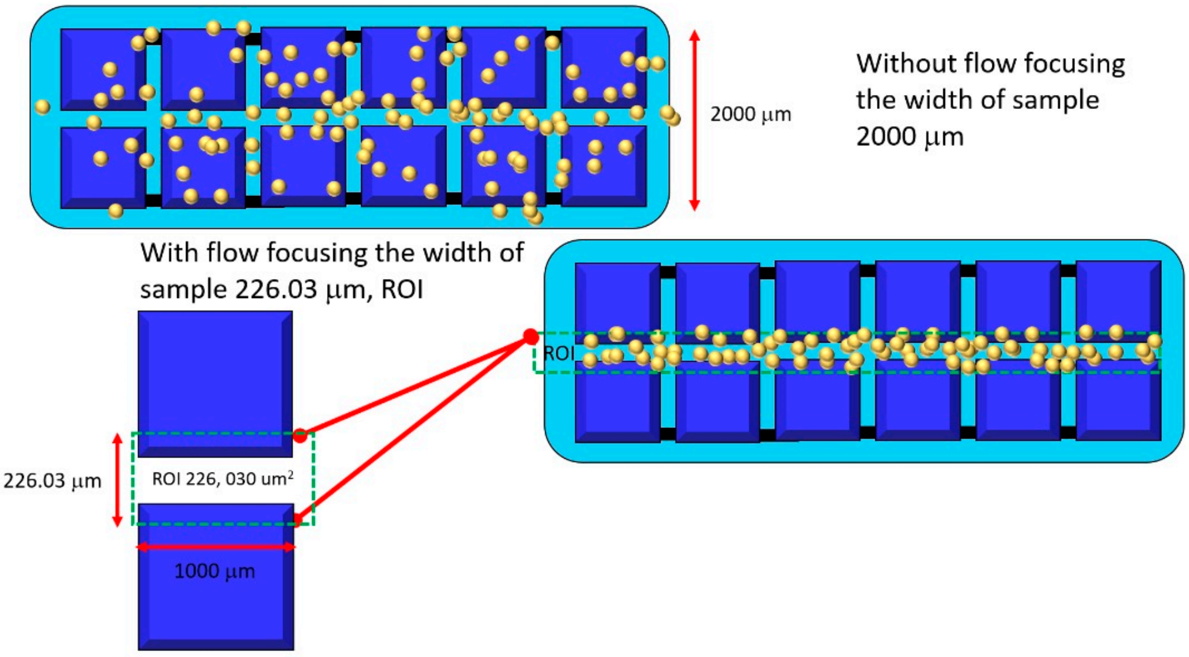

Figure S1. The width of the sample in TAMA with and without flow-focusing.

Calculation of Manipulation Efficiency (%) in table 3:

- Table 3 (In article) investigates separation efficiency by determining the area of particles covered in the ROI before and after separation using ImageJ analysis. The detailed calculation area is shown in the Figure S2 below.

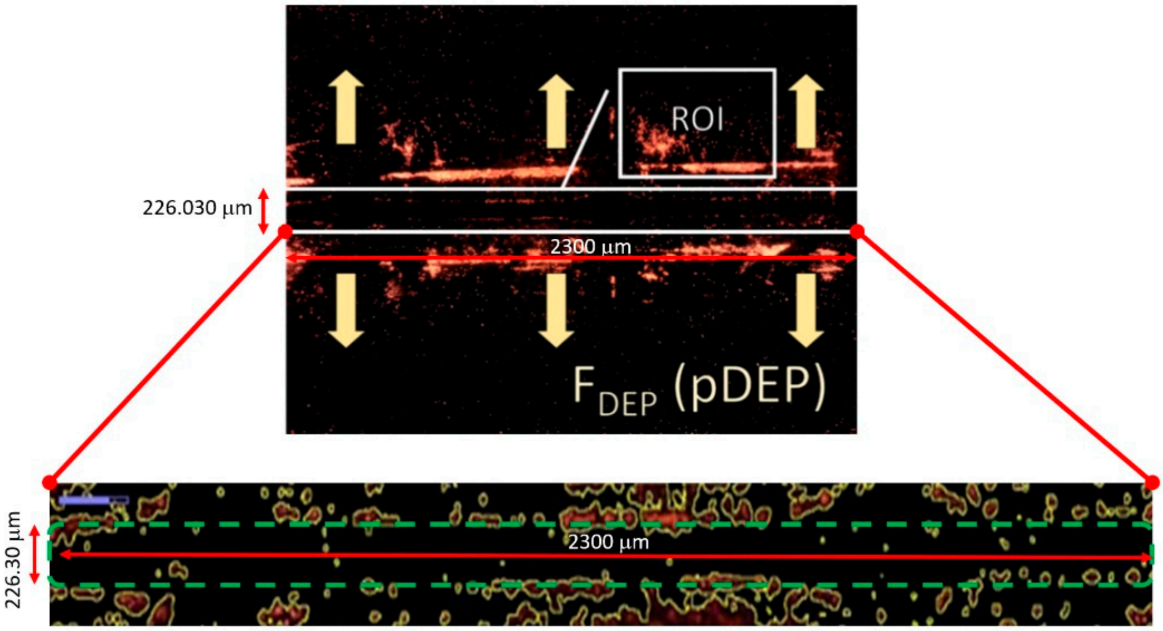

Figure S2. TAMA's manipulation efficiency calculated area.

- The ROI denotes the area in between two dotted green lines. The calculated area has a length of 2300 μm and a width of 226.03 μm approximately.

- A total of 519,869 μm^2 is covered by approximately two pairs of electrodes.

- The ImageJ calculated area after F_{DEP} without flow-focusing (519,422.8 μm^2) subjected to the particle dispersion within ROI.

((519,422.8 μm^2 - 414, 208.1 μm^2)/ (519,422.8 μm^2)) *100 = 20.3%

- After F_{DEP} with flow-focusing (517,465.7 μm^2) are subjected to the particle dispersion within ROI.

- Different particle dispersion in ROI, whereby before and after separation is divided by the total area.

((517,465.7 μm^2 -102,116.3 μm^2)/ (517,465.7 μm^2)) *100 = 80.3%
